# 

**Published:** 1887-01

**Authors:** 


### BOOK NOTICES.

We offer our greetings and good wishes to the following publications whose monthly visitations are ever welcome, upon our exchange table:

Southern California Practitioner.
Popular Science News.
Physio-Medical Journal.
Light, Heat and Power.
Progress.
American Medical Journal.
Alientist and Neurologist.
Western Agriculturist.
Western Journal of Health.
Herald of Health.
New York Medical Journal.
Sanitary Era.
Albany Medical Annals.
Materia Medical.
Educational Journal.
Journal D'Hygiene.
Journal of Reconstructives.
Therapeutic Gazette.
Medical Summary.
Medical Bulletin.

AN EPITOME OF THE NEW MATERIA MEDICA, ETC., ETC.—Embracing a complete property and dose list, designed for the special covenience of the busy physician, has just been issued by Park, Davis & Co., the well known drug firm of Detroit, Mich. The property and dose list of drugs manufactured by them covers almost everything appertaining to the curative art, drawn from nature's ample laboratory. It will be found to be a kind of ready-reckoner for physicians, besides a great convenience in filling orders upon a house that manages some how to keep on its feet without advertising in this Journal.

